# 2327. Masking without Mandates: Patient Preferences

**DOI:** 10.1093/ofid/ofad500.1949

**Published:** 2023-11-27

**Authors:** Matthew Harris, Joseph Narvaez, Annabella Salvador, Bruce Farber

**Affiliations:** Northwell Health, New Hyde Park, New York; Northwell Health, New Hyde Park, New York; Northwell Health, New Hyde Park, New York; Northshore University Hospital Northwell Health, Manhasset, New York

## Abstract

**Background:**

Masking has been a foundational component of COVID-19 transmission reduction strategies; however, mask mandates have begun to sunset. The United States Center for Disease Control and Prevention has recommended curtailing mask use in healthcare settings and guiding decisions based on community transmission rates. There is little data describing patient perspectives on the continued use of masks in clinical settings.

**Methods:**

Patient surveys were disseminated via email and SMS using census-sampling methodology to all patients with a clinical encounter within the past 3 months to gauge perceptions of the need for continued masking for patients, visitors, and medical staff in the clinical setting. Data analysis was performed using descriptive and inferential statistics. Thematic analysis was used to analyze qualitative data.

**Results:**

10,964 (5.6%) of 196,424 surveys were completed. 5,418 (49%) of respondents were age 65 and older and 7,728 (70%) were female. Respondents prefer masking at all times in the inpatient setting (Med-surg, ICU, Oncology, Pediatrics, Mother/Baby, Emergency Dept) for *clinical staff* mean(m) 61.2% (range (r) 56.5%-71.3%), *patients* m = 56.9 (r 51.5-65.4), and *visitors* m = 58.5 (r 52.8-67.0). When local transmission rates are moderate to high, preference for masking increases by 36%, 45% and 42% for *staff, patients, and visitors* respectively. Patients hospitalized for COVID-19 in the past 365 days demonstrated a preference towards masking at all times, m = 63.5, (r 59.1-73.2) and when transmission rates are elevated, m= 86.6 (r 84.5-89.5), compared to those who were not hospitalized, m = 61.7 (r 57.1-71.6) and m = 76.0 (r 72.1 – 82.7). Respondents were less likely to prefer masking in non-clinical environments (∼25%). Approximately 40% preferred mask use in the ambulatory environment for all groups.

**Conclusion:**

While mask utilization is becoming less ubiquitous in the current phase of the COVID-19 pandemic, the majority of patients prefer masks to be worn by staff, patients, and visitors in the inpatient setting. This is especially true for patients hospitalized for COVID-19 in the past year and during periods of moderate to high community transmission rates.

**Disclosures:**

**All Authors**: No reported disclosures

